# Combined full-length transcriptomic and metabolomic analysis reveals the regulatory mechanisms of adaptation to salt stress in asparagus

**DOI:** 10.3389/fpls.2022.1050840

**Published:** 2022-10-27

**Authors:** Xuhong Zhang, Changzhi Han, Yuqin Liang, Yang Yang, Yun Liu, Yanpo Cao

**Affiliations:** ^1^ Institute of Cash Crops, Hebei Academy of Agriculture and Forestry Sciences, Shijiazhuang, China; ^2^ Landscape Management and Protection Center, Shijiazhuang Bureau of Landscape Architecture, Shijiazhuang, China; ^3^ College of Biodiversity Conservation, Southwest Forestry University, Kunming, China

**Keywords:** salt stress, ion transport, metabolic adjustment, salinity tolerance, asparagus

## Abstract

Soil salinity is a very serious abiotic stressor that affects plant growth and threatens crop yield. Thus, it is important to explore the mechanisms of salt tolerance of plant and then to stabilize and improve crop yield. Asparagus is an important cash crop, but its salt tolerance mechanisms are largely unknown. Full-length transcriptomic and metabolomic analyses were performed on two asparagus genotypes: ‘jx1502’ (a salt-tolerant genotype) and ‘gold crown’ (a salt-sensitive genotype). Compared with the distilled water treatment (control), 877 and 1610 differentially expressed genes (DEGs) were identified in ‘jx1502’ and ‘gold crown’ under salt stress treatment, respectively, and 135 and 73 differentially accumulated metabolites (DAMs) were identified in ‘jx1502’ and ‘gold crown’ under salt stress treatment, respectively. DEGs related to ion transport, plant hormone response, and cell division and growth presented differential expression profiles between ‘jx1502’ and ‘gold crown.’ In ‘jx1502,’ 11 ion transport-related DEGs, 8 plant hormone response-related DEGs, and 12 cell division and growth-related DEGs were upregulated, while 7 ion transport-related DEGs, 4 plant hormone response-related DEGs, and 2 cell division and growth-related DEGs were downregulated. Interestingly, in ‘gold crown,’ 14 ion transport-related DEGs, 2 plant hormone response-related DEGs, and 6 cell division and growth-related DEGs were upregulated, while 45 ion transport-related DEGs, 13 plant hormone response-related DEGs, and 16 cell division and growth-related DEGs were downregulated. Genotype ‘jx1502’ can modulate K^+^/Na^+^ and water homeostasis and maintain a more constant transport system for nutrient uptake and distribution than ‘gold crown’ under salt stress. Genotype ‘jx1502’ strengthened the response to auxin (IAA), as well as cell division and growth for root remodeling and thus salt tolerance. Therefore, the integration analysis of transcriptomic and metabolomic indicated that ‘jx1502’ enhanced sugar and amino acid metabolism for energy supply and osmotic regulatory substance accumulation to meet the demands of protective mechanisms against salt stress. This work contributed to reveal the underlying salt tolerance mechanism of asparagus at transcription and metabolism level and proposed new directions for asparagus variety improvement.

## Introduction

Soil salinity as one of the most serious abiotic stressors can impact greatly on plant life processes and pose an enormous threat to crop production (Rengasamy, 2010; Duarte et al., 2013; Munns and Gilliham, 2015). To improve the utilization of saline land in the world, the cultivation of plants with tolerance to salt may be a very effective strategy. So it is particularly important to carry out researches on the identification of salt-tolerant plants and the exploration on underlying tolerance mechanisms for further plant variety improvement (Li et al., 2022).

High sodium concentrations in the soil limit water uptake and nutrient absorption (Hussain et al., 2021) and cause ionic toxicity, osmotic stress, and oxidative stress (Zhang et al., 2019a). To reduce the possible threats of above stresses, most of plants have developed mature mechanisms to response to external signals (Hasegawa et al., 2000), which may involve every step in the signaling pathways, such as Ca^2+^ and plant hormone pathways, and subsequent adaptive responses, including the regulation of ion balance, osmotic pressure, and plant development and growth (Van Zelm et al., 2020; Zhang et al., 2021). After the initial perception of excessive Na^+^, the Ca^2+^ signal and plant hormones, as important intracellular secondary messengers, are usually triggered. In calcium signaling pathway, some kinases participate in decoding Ca^2+^ signals and thus cause a variety of cellular responses to sodium, which involving calcium-dependent protein kinases (CDPKs), calcineurin B-like proteins (CBLs), and CBL-interacting protein kinases (CIPKs) (Manishankar et al., 2018; Amirbakhtiar et al., 2019; Van Zelm et al., 2020). Plant hormones are important endogenous signals for regulating plant growth and development under both normal and stressed conditions (Van Zelm et al., 2020). Importantly, roots, as the frontline tissue exposed to soil salinity, need to maintain the growth and the absorption of water and nutrients for adapting the stressed environment (Ouyang et al., 2007). The developmental modifications to root system architecture is vital for tolerance to high soil salinity and it strongly depends on auxin (IAA) signaling (He et al., 2018; Korver et al., 2018). Recent evidence suggested important contributions of IAA metabolism and transport on eventual IAA accumulation and signaling patterns (Korver et al., 2018). It was reported that the Group II members of the GRETCHEN HAGEN 3 (GH3) gene family mediate IAA metabolic inactivation through catalysing the conjugation of IAA to amino acids (Staswick et al., 2002; Staswick et al., 2005), the gh3oct mutant plants, in which the entire group II GH3s were knocked out, showed a more branched root system and tolerance to salinity (Casanova-Sáez et al., 2022). Additionally, the long distance transport of IAA from shoot to root and its movement among local cells are necessary for root growth as well as adaption to external environments (Korver et al., 2018; Van Zelm et al., 2020). In these processes, ATP binding cassette B/P-glycoprotein/Multidrug-resistance (ABCB/PGP/MDR), auxin resistant 1/like aux 1 (AUX/LAX), and pin-formed (PIN) contribute to IAA distribution to mediate plant growth responses and thus adaption to salt stress (Galvan-Ampudia et al., 2013; Yue et al., 2015; Van den Berg et al., 2016). Abscisic acid (ABA) signaling plays a crucial role in the quiescence of root growth induced by salt stress, and its concentration is reduced during the recovery phase (Duan et al., 2013; Duan et al., 2015; Van Zelm et al., 2020). Besides to modulate root growth, ABA signaling also mediates several other processes, such as gene transcription, stomatal closure, ion transport, and reactive oxygen species (ROS) production, through phosphorylating various downstream targets by sucrose non-fermenting 1-related protein kinases (SnRKs) (Van Zelm et al., 2020). In addition, the accumulation of primary metabolites, including amino acids, sugars, and polyols, was reported to contribute to the plant salt tolerance (Ashraf and Foolad, 2007; Li et al., 2011). Sugar metabolism and amino acid metabolism are crucial to providing energy and osmotic regulatory substances in response to salt stress (Liu et al., 2020a).

Asparagus (Asparagus officinalis L.), a kind of perennial herb, has important edible, medicinal, as well as economic value. Asparagus has moderate salinity tolerance, whereas depending on the genotypes, it exhibits different salt tolerances in heavy saline soil (Zhang et al., 2020; Gao et al., 2021). But less is known for us about the salt tolerance mechanism of asparagus at molecular level. In a previous study, we preliminarily investigated the global gene transcription response mechanism of leaves of asparagus variety ‘No. 08-2’ to salinity stress using the Illumina HiSeq™ 2500 sequencing platform, suggesting the crucial roles of ion transport, ROS metabolism, and carbon metabolism in response to salinity stress (Zhang et al., 2020). There are still few works on exploring the response of asparagus roots to salt stress. Roots are the key plant tissue exposed to soil salinity and play crucial roles in the uptake of nutrients and water, the secretion of organic acids and enzymes, and the production of hormones (Ouyang et al., 2007; Zhai et al., 2013). Not only the widely changed gene expression, the adjusted metabolite accumulation in response to salt stress also takes part in regulating plant growth and its adaptation to salt stress (Huang et al., 2018; Zhang et al., 2019a; Liu et al., 2020a). However, there is still a lack of research on the asparagus metabolism response to salt stress and its underlying connection with the gene response to salt stress is also not clear. While, these are required for facilitating the breeding of salt-tolerance asparagus.

Using an integrated transcript/metabolite approach, a better understanding of salt tolerance mechanisms in many species (Ma et al., 2019; Zhang et al., 2019a; Liu et al., 2020a), has been built. In such reports, second-generation sequencing technology (SGS) has been widely employed for transcriptome analysis to screen differential gene expression under salt stress. With the development of sequencing technology, Oxford Nanopore Technologies (ONT) MinION, a kind of third-generation sequencing technology (TGS), shows more advantages in several respects compared with SGS. Specifically, ONT can achieve full-length transcriptome sequencing, quantify at isoform level, and identify complex structures of gene (Zhao et al., 2019; Zhang et al., 2021), and thus also opens a new point for exploring plant tolerance mechanisms.

Here, the transcriptome and metabolome of the seedling roots of ‘jx1502’ and ‘gold crown’ were analyzed three days after salt treatments, in which the ONT MinION platform and widely targeted metabolomic analysis were used. Through the analysis of transcriptome and metabolome data between the two contrasting genotypes, we sought to reveal the crucial salt response genes and metabolites and their potential links.

## Materials and methods

### Plant materials and salt treatments

Asparagus genotypes ‘jx1502’ (a salt-tolerant) and ‘gold crown’ (a salt-sensitive) were selected in this work (Gao et al., 2021). For the acquisition of experimental seedlings, the method in our previous study was applied (Zhang et al., 2020). Then almost 60-day-old seedlings accepted salt stress treatment with 200 ml solution of 100 mM NaCl, while the controls were irrigated with distilled water. After three days of treatment, total 12 root samples of two asparagus genotypes for each treatment with three biological replicates were collected.

### RNA extraction, cDNA library construction, and sequence assay

The RNA extraction, cDNA library construction, and sequence assay for 12 root samples were conducted by Biomarker Biotechnology Corporation (Beijing, China). The concrete methods were same with those described in Zhang et al’s study (2021). In these processes, full-length cDNA libraries were constructed and Oxford Nanopore Technologies (ONT) MinION sequencer was used for sequence assay

### Transcriptome analysis

Transcriptome analysis referred to the methods of Zhang et al. (2021). Firstly, through the filter of low quality and short read length reads and the elimination of ribosomal RNA from raw reads, clean reads were obtained and subsequently full-length transcripts were detected. Then the reference transcriptome was constructed for full-length reads mapping and consequence analysis through combining the final transcripts after removing redundancy and the known transcripts of the genome (GCA_001876935.1, 2017). For quantitative analysis, the reads per gene per 10,000 mapped reads were used to estimate expression levels. The differential expression analysis between two treatments were conducted by DESeq2 R package. Genes with the fold change ≥ 2 and a P value< 0.01 were appointed as differentially expressed genes (DEGs). Among the identified DEGs through transcriptome analysis, 9 DEGs were selected to confirm their expression patterns using quantitative real-time PCR (qRT-PCR) analysis, with 3 technical replicates for each sample of 3 biological replicates, following Zhang et al. (2021). The specific primers for asparagus are shown in **Supplementary Table 1**.

In addition, Kyoto Encyclopedia of Genes and Genomes (KEGG) and Gene Ontology (GO) enrichment analyses for DEGs were performed in several key comparisons. Alternative splicing (AS) events were identified using the AStalavista tool. Transcription factors (TFs) were identified with iTAK.

### Sample preparation, metabolite extraction, and data analysis

The root samples that we collected in advance and stored at -80°C were used for metabolite analysis. The metabolite extraction and its identification and quantification analysis were carried out by Wuhan MetWare Biotechnology Co., Ltd. (http://www.metware.cn). Briefly, the freeze-dried roots were crushed, and 100 mg was used for extraction with 70% aqueous methanol of 1.2 ml at 4°C for overnight. Following centrifugation, extracts were absorbed and filtered before ultra-performance liquid chromatography-mass/mass spectrometry (UPLC-MS/MS) (UPLC, Shim-pack UFLC SHIMADZU CBM30A system, http://www.shimadzu.com.cn/; MS, Applied Biosystems 6500 Q TRAP, http://www.appliedbiosystems.com.cn/) analysis referring to the procedures used in others’ studies (Yuan et al., 2018; Zhang et al., 2019b). With the metabolite information in MetWare Database and public database, the qualitative analysis of metabolite was conducted in accordance with the secondary spectrum information. In addition, quantitative analysis was performed with the multiple reaction monitoring mode. Partial least squares discriminant analysis was used for metabolite information mining. Metabolites with VIP (variable importance in projection) ≥ 1 and absolute Log_2_FC (fold change) ≥ 1 were appointed as differentially accumulated metabolites (DAMs). The KEGG Compound database (http://www.kegg.jp/kegg/compound/) and KEGG Pathway database (http://www.kegg.jp/kegg/pathway.html) were used for the annotation of identified metabolites.

## Results

### Transcriptome analysis for ‘jx1502’ and ‘gold crown’

The transcriptomes of ‘jx1502’ and ‘gold crown’ under different treatments obtained by using ONT were compared (**Supplementary Table 2**). Through transcriptome sequencing, there were 42.52 million clean reads generating from 12 asparagus roots samples. And the base calling accuracy was all more than 90.00% for every library. The percentages of full-length reads between 77.00% and 80.39% were obtained.

### Identification of DEGs under salt stress

**Figure 1:**
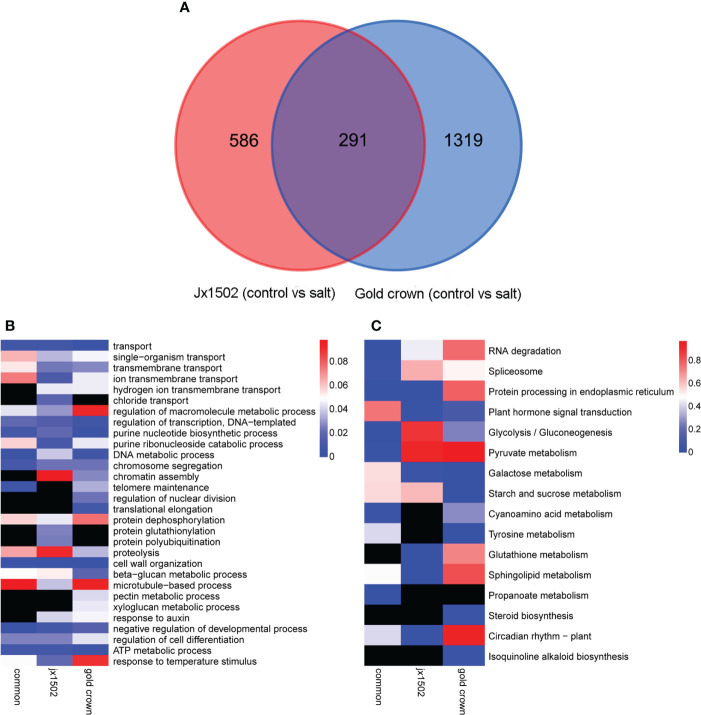
**(A)** Venn diagram showing specific and common differentially expressed genes in ‘jx1502’ and ‘gold crown’ under salt stress. **(B, C)** Significantly enriched GO terms and KEGG pathways with ‘jx1502’-specific, ‘gold crown’-specific, and common DEGs under salt stress. The color scale represents the p-value of the enriched term and pathway. The black boxes indicate that there are no DEGs that were enriched in the corresponding term or pathway.

In total, 877 and 1610 DEGs were identified in ‘jx1502’ and ‘gold crown’ under salt stress compared with the distilled water treatment (control), including 519 and 483 upregulated DEGs, respectively, and 358 and 1127 downregulated DEGs, respectively. There were many more DEGs in ‘gold crown’ than in ‘jx1502,’ indicating that the salt-sensitive genotype responds to salinity stress in a wider range. For ‘jx1502,’ the number of upregulated DEGs was much larger than that of downregulated DEGs, while more than 2 times the number of DEGs was downregulated than upregulated in ‘gold crown,’ which revealed their diverse response patterns in relation to corresponding adaptive mechanisms. For further exploring the relationships of DEGs between the two genotypes in response to salt stress, a Venn diagram was constructed (**Figure 1A**). Among these, 291 DEGs were common in both genotypes. There were also 586 and 1319 specific DEGs in ‘jx1502’ and ‘gold crown,’ respectively.

GO and KEGG enrichment analyses were performed using common and specific DEG sets (**Figures 1B, C**). GO enrichment analysis revealed that “transport” was significantly enriched in most DEGs in all three DEG sets, suggesting the crucial role of this term in the roots’ response to salt stress. Some DEGs involved in ion transport from both genotype-specific sets were significantly enriched in “ion transmembrane transport” and “hydrogen ion transmembrane transport,” while “chloride transport” was only significantly enriched in ‘jx1502’-specific DEGs. Biological processes related to genetic information processing and protein metabolism were also significantly enriched with a lot of DEGs. In addition, there were some significantly enriched terms related to cell wall metabolism. For example, “cell wall organization” and “beta-glucan metabolic process” were significantly enriched with common DEGs and several genotype-specific DEGs. In addition, “microtubule-based process” was only significantly enriched with ‘jx1502’-specific DEGs, and “xyloglucan metabolic process” and “pectin metabolic process” were only significantly enriched in the ‘gold crown’ genotype. Moreover, “response to auxin” was significantly enriched in both genotype-specific DEG sets. KEGG enrichment analysis revealed that the pathways related to RNA degradation, spliceosome, and protein processing, as well as sugar and amino acid metabolism, were significantly enriched with common DEGs. In addition, ‘jx1502’-specific DEGs were significantly enriched in “Glutathione metabolism” and “Plant hormone signal transduction”, and ‘gold crown’-specific DEGs were significantly enriched in “Tyrosine metabolism” and “Starch and sucrose metabolism”.

### DEGs involved in ion transport, plant hormone signal transduction, and cell division and growth

**Figure 2:**
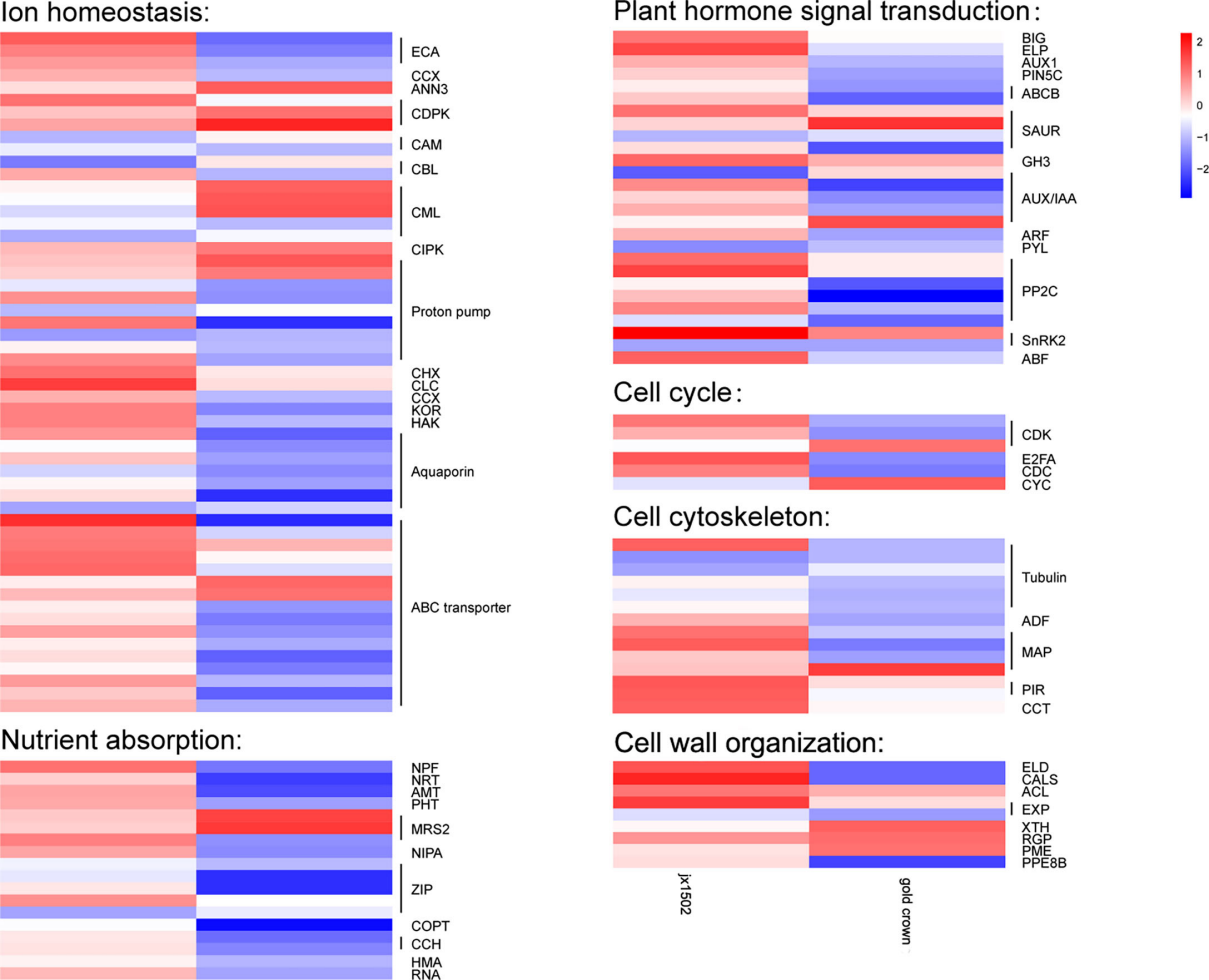
Heatmap of the expression patterns of the differentially expressed genes related to ion homeostasis, nutrient absorption, plant hormone signal transduction, the cell cycle, the cell cytoskeleton, and cell wall organization in ‘jx1502’ (left) and ‘gold crown’ (right) under salt stress. The color scale represents the log2 value of the fold change.

In this study, between two asparagus genotypes, several genes encoding for various transporters and proteins related to ion transport and homeostasis were differentially regulated under salt stress (**Figure 2** and **Supplementary Table 3**). In ‘jx1502,’ we found 1 calcium-transporting ATPase (ECA) gene, 1 calcium-dependent protein kinase (CDPK) gene, 1 proton pump gene, 1 cation/H^+^ antiporter (CHX) gene, and 1 chloride channel (CLC) gene were upregulated, while 1 calmodulin (CAM) gene, 1 calcineurin B-like protein (CBL) gene, 1 calcium-binding protein (CML) gene, and 2 Proton pump genes were downregulated under salt stress. In ‘gold crown,’ 1 annexin D3 (ANN3) gene, 2 CDPK genes, 3 CML genes, 1 CBL-interacting protein kinase (CIPK) gene, and 2 proton pump genes were upregulated, while 4 ECA genes, 1 cation/calcium exchanger (CCX) gene, 1 CAM gene, 1 CML gene, 6 proton pump genes, 1 potassium channel (KOR) gene, and 1 potassium transporter (HAK) gene were downregulated under salt stress. Aquaporins play key roles in responding to salt stress. In ‘jx1502,’ only one tonoplast intrinsic protein (TIP) gene was downregulated under salt stress. In ‘gold crown,’ 1 nodulin-like intrinsic protein (NIP) gene, 1 TIP gene, and 4 plasma membrane intrinsic protein (PIP) genes were downregulated. In response to salt stress, 5 ABC transporter genes were upregulated in ‘jx1502,’ while 2 ABC transporter genes were upregulated and 10 ABC transporter genes were downregulated in ‘gold crown.’ Several important genes for the transport of nutrition ions also showed differential response patterns to salt stress between the two genotypes. In ‘jx1502,’ 1 protein NRT1/PTR FAMILY (NPF) gene was upregulated under salt stress. In ‘gold crown,’ 1 NPF gene, 1 high-affinity nitrate transporter (NRT) gene, 1 ammonium transporter (AMT) gene, 1 phosphate transporter (PHT) gene, 2 magnesium transporter (MRS2, NIPA) genes, 3 Zn^2+^, Fe^2+^, Mn^2+^ transporter (ZIP) genes, 1 copper transporter (COPT) gene, 2 copper transport protein (CCH) genes, and 2 copper-transporting ATPase (HMA, RNA) genes were downregulated under salt stress.

In our study, several DEGs related to IAA and ABA signal transduction were identified, which exhibited differential responses to salt stress in ‘jx1502’ and ‘gold crown’ (**Figure 2** and **Supplementary Table 3**). For IAA transport, under salt stress, 1 auxin transport protein BIG gene, 1 elongator complex protein (ELP) gene, 1 auxin-responsive protein SAUR gene, and 1 indole-3-acetic acid-amido synthetase (GH3) gene were upregulated, and 1 auxin-responsive protein SAUR gene and 1 auxin-induced protein/Auxin-responsive protein (AUX/IAA) were downregulated in ‘jx1502,’ while 1 auxin-responsive protein SAUR gene, and 1 AUX/IAA gene were upregulated, and 1 auxin transporter-like protein gene (AUX), 1 auxin efflux carrier component 5c gene (PIN5C), 2 ABC transporter B family member (ABCB) genes, 1 auxin-responsive protein SAUR gene, 3 AUX/IAA genes, and 1 auxin response factor (ARF) gene were downregulated in ‘gold crown.’ As for ABA signal transduction, 2 protein phosphatase 2C (PP2C) genes, 1 snf1-related kinase 2 gene (SnRK2), and 1 ABA responsive element binding factor gene (ABF) were upregulated in ‘jx1502.’ One abscisic acid receptor (PYL) gene and 1 SnRK2 gene were downregulated in ‘jx1502,’ while 4 PP2C genes and 1 SnRK2 gene were downregulated in ‘gold crown.’

In this study, cell cycle-related genes presented different response profiles to salt stress between ‘jx1502’ and ‘gold crown.’ In ‘jx1502,’ 1 cyclin-dependent kinase (CDK) gene and 1 transcription factor E2FA were upregulated. In ‘gold crown,’ 1 cyclin (CYC) gene and 1 CDK gene were upregulated, 1 cell division cycle protein (CDC) gene, 1 CDK gene, and 1 transcription factor E2FA were downregulated. For cell growth, 1 tubulin gene, 2 microtubule-associated protein (MAP) genes, 2 protein PIR genes, and 1 T-complex protein (CCT) gene were upregulated in ‘jx1502,’ while 5 tubulin genes, 1 actin-depolymerizing factor (ADF) gene, and 2 MAP genes were downregulated in ‘gold crown.’ In addition, several DEGs encoding cell wall-related proteins were found to be differentially regulated between two asparagus genotypes in response to salt stress. In ‘jx1502,’ 1 glycosyltransferase-like KOBITO (ELD) gene, 1 callose synthase (CALS) gene, 1 thermospermine synthase ACAULIS5 (ACL) gene, and 1 expansin (EXP) gene were upregulated. In ‘gold crown,’ 1 xyloglucan endotransglucosylase/hydrolase (XTH) gene, 1 UDP-arabinopyranose mutase (RGP) gene, and 1 pectinesterase (PME) gene were upregulated, while 1 ELD gene, 1 CALS gene, 1 EXP gene, and 1 pectinesterase PPE8B gene were downregulated (**Figure 2** and **Supplementary Table 3**).

### Identification of TFs and AS events

**Figure 3:**
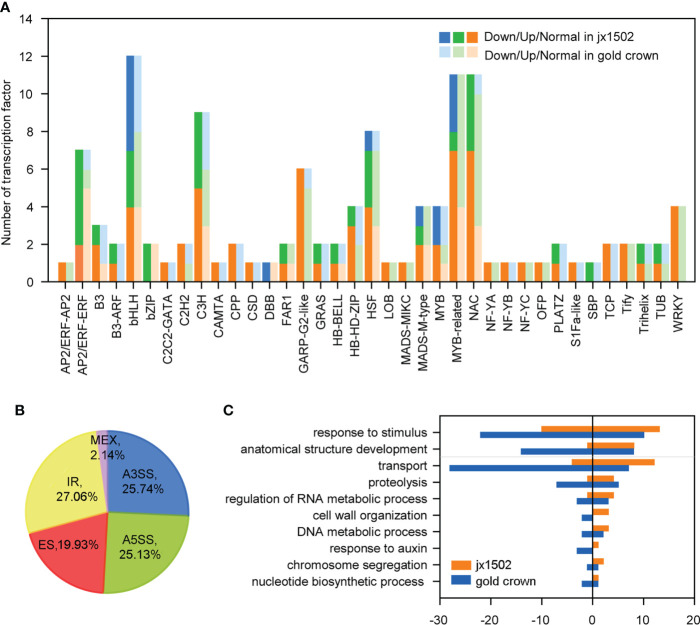
**(A)** Number and expression profile of TFs identified in ‘jx1502’ and ‘gold crown’ under salt stress. **(B)** Distribution of five types AS events in 12 libraries. **(C)** GO annotation of biological process for DAS-DEGs under salt stress. The positive and negative numbers represent the numbers of upregulated and downregulated DAS-DEGs under salt stress, respectively.

In this study, total 120 TFs were differentially expressed under salt stress in two genotypes. There were 46 and 89 differentially expressed TFs that were identified in ‘jx1502’ and ‘gold crown,’ respectively, and mainly belonged to the TF families MYBs and MYB-related, bHLH, NAC, C3H, and HSF (**Figure 3A** and **Supplementary Table 4**). As shown in **Figure 3A**, the response pattern of the bHLH, NAC, HSF, and C3H family members was basically the same in both genotypes, with about half or more members upregulated. In particular, MYB and MYB-related family members were mainly upregulated in ‘gold crown’ but downregulated in ‘jx1502.’

Additionally, there were 4712 AS events in all 12 libraries, which were identified from 3443 genes (**Supplementary Table 5**). Five kinds of AS events were found, among which intron retention IR events (27.06%) were the most, common followed by alternative 3′ splice site A3SS (25.74%), alternative 5′ splice site A5SS (25.13%), exon skipping ES (19.93%), and mutually exclusive exon MXE (2.14%) (**Figure 3B**). In addition, we focused on DEGs with different AS events (DAS-DEGs) in both genotypes. In total, 172 DAS-DEGs were detected in ‘jx1502,’ with 109 upregulated and 63 downregulated genes. In addition, 290 DAS-DEGs were detected in the salt-sensitive genotype, with 110 upregulated and 180 downregulated genes. Based on GO analysis, the DAS-DEGs in the two genotypes were significantly enriched to some common terms, including “transport,” “regulation of RNA metabolic process,” “cell wall organization,” “DNA metabolic process,” “chromosome segregation,” and “nucleotide biosynthetic process” (**Figure 3C**). “Proteolysis” and “response to auxin” were significantly enriched only in ‘gold crown.’ In addition, there were many DAS-DEGs in both genotypes enriched in “response to stimulus” and “anatomical structure development” that were closely related to salt tolerance.

### QRT-PCR validation

Nine DEGs in ‘jx1502’ and ‘gold crown’ under salt stress were randomly selected for qRT-PCR validation. The qRT-PCR expression trend of these nine genes was highly consistent with the RNA-seq results (**Supplementary Figure 1**).

### Metabolome reprogramming under salt treatment in ‘jx1502’ and ‘gold crown’

**Figure 4:**
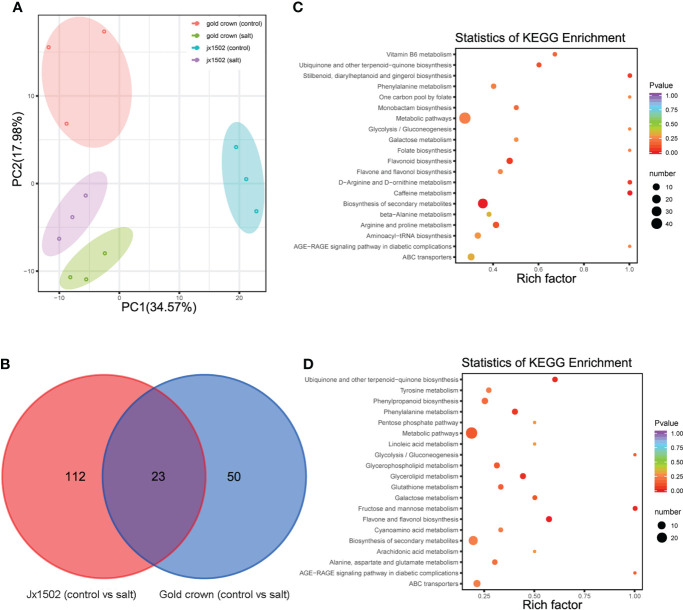
**(A)** PCA clustering based on metabolome data in ‘jx1502’ and ‘gold crown’ under salt stress. **(B)** Venn diagram showing specific and common metabolites that are differentially regulated in ‘jx1502’ and ‘gold crown’ under salt stress. **(C)** The KEGG pathway enrichment scatter map based on differentially accumulated metabolites in ‘jx1502’ under salt stress. **(D)** The KEGG pathway enrichment scatter map based on differentially accumulated metabolites in ‘gold crown’ under salt stress.

For the aim to explore the difference in salt tolerance between ‘jx1502’ and ‘gold crown’ at the metabolic level, the analysis of metabolite profiles in the roots of both genotypes was conducted by using UPLC-MS/MS. A total of 491 metabolites were identified, including sugars, amino acids, organic acids, phenolic acids, lipids, and flavonoids. Principal component analysis (PCA) presented a clear separation between the distilled water treatment and salt treatment in both ‘jx1502’ and ‘gold crown,’ as well as a separation of genotypes under both control and salt treatment in the first two principal components (**Figure 4A**).

Compared with the control, 185 metabolites were identified to be significantly changed in ‘jx1502’ and ‘gold crown’ after salt stress. Among these metabolites, 135 DAMs and 73 DAMs were identified in ‘jx1502’ and ‘gold crown’ under salt stress compared with the control, including 32 and 40 increased DAMs, respectively, and 103 and 33 decreased DAMs, respectively. In the intersection analysis (**Figure 4B**), only 23 salt-responsive metabolites were identified across the different genotypes. Additionally, 112 and 50 metabolites were associated with salt stress in ‘jx1502’ and ‘gold crown,’ respectively. Through metabolic pathway mapping, DAMs identified from the two genotypes were both highly enriched in pathways mainly involved in amino acid metabolism, sugar metabolism, biosynthesis of secondary metabolites, and glycerolipid metabolism (**Figures 4C, D**), indicating their important roles contributing to salt tolerance of asparagus. Furthermore, by checking the detailed content adjustment of these metabolites, many showed different adjustment patterns between ‘jx1502’ and ‘gold crown’ under salt stress conditions (**Supplementary Table 6**). For instance, sugars, and polyols (6 out of 7) were obviously upregulated in ‘jx1502’ after salt stress, such as glucose, melibiose, and turanose, while those (8 out of 9) in ‘gold crown,’ such as glucose, melezitose, panose, and sorbitol, were mainly decreased. Fifteen organic acids, including citrate, succinate, fumaric acid, aminobutyric acid, homoserine, and pipecolinic acid that were upregulated, were regulated by ‘jx1502’ under salt treatment, whereas in ‘gold crown,’ there were only two organic acids (phosphoenol-pyrurate and citrate) in response to salt stress that showed a decrease. In addition, there were 6 and 4 amino acids that were increased in ‘jx1502’ and ‘gold crown,’ respectively, among which only one amino acid (asparagine) was common in the two genotypes and showed greater changes in ‘jx1502.’ In addition, lipids, phenolic acids, and flavonoid metabolites also showed obvious differences between the two genotypes in response to salt stress.

### Association analysis of transcriptomic and metabolomic

To further explore the intrinsic mechanism of asparagus salt tolerance, it may be a crucial step to integrate the connection between salt-responsive genes and metabolites. In our study, a lot of DEGs and DAMs were widely annotated to several sugar metabolism and amino acid metabolism-related pathways.

### Sugar metabolism analysis of salt-stressed asparagus

**Figure 5:**
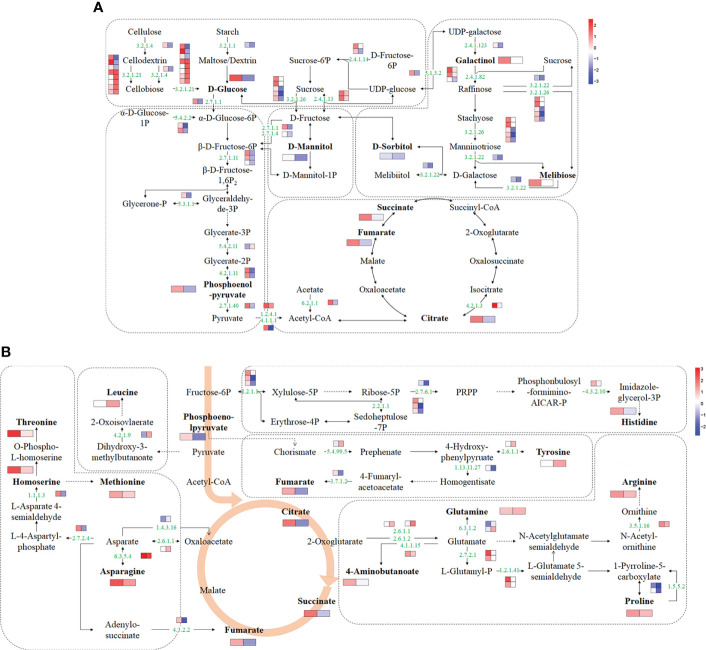
Schematic networks of the sugar metabolism **(A)** and amino acid metabolism **(B)**. The metabolites in bold were identified as differential metabolites in this study. The numbers in green are enzyme identifier numbers. Omitted metabolic processes are represented by dashed lines. The icons with two blocks that are colored in a gradient color—from low (blue) to high (red) for each DEG (small blocks) or differential metabolite (large blocks)—are the log of fold change (log_2_FC) in ‘jx1502’ and ‘gold crown’ under salt stress.

Based on transcriptome and metabolome data, a schematic sugar metabolism network was built with DAMs and DEGs. In total, 9 differential metabolites and 41 DEGs from 5 pathways (starch and sucrose metabolism, glycolysis/gluconeogenesis, TCA cycle, galactose metabolism, fructose, and mannose metabolism) related to sugar metabolism were included in the network (**Figure 5A** and **Supplementary Table 7**). Among the metabolites, glucose and citrate were upregulated in ‘jx1502’ but decreased in ‘gold crown.’ Specifically, galactinol, melibiose, fumaric acid, and succinate were only increased in ‘jx1502.’ Sorbitol, mannitol, and phosphoenol-pyrurate were specifically decreased in ‘gold crown.’ At the transcription level, the upregulated expression of most DEGs in ‘jx1502’ and the downregulated expression in ‘gold crown’ may contribute to the differences in their metabolite content and salt tolerance. For instance, in starch and sucrose metabolism, glucose accumulation can be regulated by the hydrolysis of sucrose, starch, or cell wall polysaccharides. Our transcriptome data showed that DEGs encoding beta-fructofuranosidase (EC:3.2.1.26) and sucrose synthase (EC:2.4.1.13), which play key roles in sucrose hydrolysis, were upregulated in ‘jx1502’ but downregulated or remained consistent in ‘gold crown.’ Similarly, the DEGs encoding endoglucanase (EC:3.2.1.4) and beta-glucosidase (EC:3.2.1.21), which catalyze the key steps of cellulose hydrolysis, were upregulated in ‘jx1502,’ whereas about half were downregulated in ‘gold crown.’ Moreover, the expression level of a DEG encoding pectinesterase (EC:3.2.1.11) for starch hydrolysis was also downregulated in ‘gold crown.’ The response pattern of the above DEGs may contribute to glucose accumulation in ‘jx1502’ but decrease in ‘gold crown.’ In galactose metabolism, the increase of galactinol and melibiose in ‘jx1502’ could be primarily attributed to the induced expression of genes encoding their synthesis-related enzymes, including sucrose synthase (EC:2.4.1.13), raffinose synthase (EC:2.4.1.82), and beta-fructofuranosidase (EC:3.2.1.26). The related DEGs in this pathway were downregulated in the ‘gold crown,’ and the content of galactinol and melibiose was also decreased accordingly to a certain degree. In addition, most of the DEGs involved in fructose and mannose metabolism, glycolysis/gluconeogenesis, and the TCA cycle were also upregulated in ‘jx1502,’ while downregulated in ‘gold crown,’ which may contribute to the corresponding adjustment of polyols and organic acids in two genotypes.

### Amino acid metabolism analysis of salt stress

For amino acid metabolism, 9 differential amino acids and 23 DEGs were involved in the schematic network (**Figure 5B** and **Supplementary Table 7**). The amino acid content in response to salt stress was significantly increased in both genotypes, but the types of amino acids, except for asparagine, were different in the two genotypes. Corresponding to the accumulation of amino acids under salt stress, most DEGs involved in their biosynthesis were upregulated. In detail, the asparagine content was increased in both genotypes, especially in ‘jx1502,’ which was consistent with the upregulated expression of a gene encoding asparagine synthase (EC: 6.3.5.4) that catalyzed the synthesis of asparagine from aspartate. In ‘jx1502,’ leading to the strong salt-induced accumulation of threonine, methionine, histidine, arginine, and proline, most DEGs involved in the biosynthesis of these amino acids were upregulated under salt stress, such as those encoding homoserine dehydrogenase (EC: 1.1.1.3), glutamine amidotransferase (EC: 4.3.2.10), acetylornithine deacetylase (EC: 3.5.1.6), and delta-1-pyrroline-5-carboxylate synthetase (EC: 2.7.2.1; EC: 1.2.1.41). Moreover, the downregulated expression of a gene encoding proline dehydrogenase (1.5.5.2), which is involved in the degradation of proline, may further promote proline accumulation in ‘jx1502.’ Although the same degradation genes were also downregulated in the ‘gold crown’ without significantly changing the expression of genes related to synthesis, the increase in proline content in ‘gold crown’ was not obvious. For tyrosine accumulation in ‘gold crown,’ given the greatly downregulated expression of genes encoding the crucial tyrosine degradation enzymes, including 4-hydroxyphenylpyruvate dioxygenase (EC: 1.13.11.27) and fumarylacetoacetase (EC: 3.7.1.2), and the upregulation of related genes in the synthesis pathway, the increased accumulation of tyrosine in ‘gold crown’ may be achieved by reduced degradation combined with increased biosynthesis. In addition, the upregulated expression of genes encoding glutamine synthetase (EC:6.3.1.2) and dihydroxy-acid dehydratase (EC: 4.2.1.9) in ‘gold crown’ under salt stress may contribute to the accumulation of glutamine and leucine in it.

Besides amino acids, ‘jx1502’ specifically accumulated some organic acids (aminobutanoste, homoserine, and those in the TCA cycle) in this network, which were also closely related to the upregulation of their synthesis-related genes. For example, salt-induced expression of the gene encoding glutamate decarboxylase (EC: 4.1.1.15) promoted the synthesis of aminobutanoste from glutamate, and the gene encoding homoserine dehydrogenase (EC: 1.1.1.3) promoted the synthesis of homoserine from L-Asparate 4-semialdehyde. In summary, the integration of both omics data suggested that the accumulation of different amino acids and organic acids may be a positive feature of withstanding salt stress.

## Discussion

Soil salinity is a major abiotic stress that seriously constrains plant growth and development (Rengasamy, 2010; Duarte et al., 2013; Munns and Gilliham, 2015). To adapt to salt stress, several plants have evolved complex mechanisms to combat salinized environments. Here, the full-length transcriptome and metabolome of asparagus were comprehensively analyzed after salt stress treatment. As a result, the extensive transcriptional and metabolic adjustments in asparagus were triggered by salt stress and were mainly involved in ion transport, root growth, and sugar and amino acid metabolism.

### Ion transport systems are more active in ‘jx1502’ under salt stress based on transcriptome analysis

Salt stress usually causes excess Na^+^ accumulation and thus plant cell toxicity. In this process, K^+^ uptake and its related physiological functions were significantly inhibited (Chen et al., 2011; Benito et al., 2014). So, a relatively balanced cytosolic K^+^/Na^+^ ratio may be regarded as a key salt tolerance trait for plants (Van Zelm et al., 2020). Proton pumps build up the proton-motive force necessary for ion transport and Na^+^ homeostasis (Van Zelm et al., 2020). Most DEGs encoding proton pumps, including P-type ATPase and pyrophosphate-energized membrane proton pumps, that fuel the Na^+^ efflux were downregulated by salt stress in ‘gold crown.’ In addition, the gene encoding HAK, which mainly mediates K^+^ uptake in roots (Nieves-Cordones et al., 2016), and CCX, which serves as a Na^+^/K^+^ exchanger in the tonoplast to perform Na^+^ sequestration in the vacuole and K^+^ accumulation in the cytoplasm (Chen et al., 2011), were also downregulated in ‘gold crown’ while remaining constant in ‘jx1502.’ These were disadvantageous for ‘gold crown’ to maintain K^+^/Na^+^ homeostasis under salt stress. In contrast to ‘gold crown,’ some more positive responses of transporter DEGs to salt stress in ‘jx1502’ may contribute to effective maintenance of K^+^/Na^+^ homeostasis. For example, CHXs that mediate K^+^ transport in response to salt stress (Jia et al., 2018; Isayenkov et al., 2020) were upregulated in ‘jx1502’ while remaining constant in ‘gold crown.’ Another obviously different response between the two genotypes was shown in the regulation of an ABC transporter that is also involved in the modulation of K^+^/Na^+^ homeostasis (Mahajan et al., 2017). In ‘jx1502,’ five DEGs encoding ABC transporters were upregulated, whereas 10 out of 12 DEGs encoding ABC transporters were downregulated in ‘gold crown.’ These results indicate that the more active ion transport systems of ‘jx1502’ may contribute to its superior K^+^/Na^+^ homeostasis and higher salt tolerance than ‘gold crown.’ Additionally, large amounts of Cl^-^ accumulation induced by salt stress also have many adverse effects on plants (Liu et al., 2020b). Studies showed that several CLC genes take part in plant salt tolerance and speculated that they play roles by mediating Cl^-^ transport across the tonoplast (Nguyen et al., 2016). Therefore, the specifically upregulated expression of the CLC gene in ‘jx1502’ under salt stress may also contribute to its higher salt tolerance compared with ‘gold crown.’

However, salt usually affects plant uptake and nutrient transport (e.g., ${\text{NO}}_{3}^{-}$ , ${\text{NH}}_{4}^{+}$ , ${\text{PO}}_{4}^{3-}$ , Mg^2+^, Zn^2+^, Fe^2+^, and Mn^2+^) (Tuna et al., 2008) and thus limits plant growth. In our study, several important genes for the transport of nutrition ions, such as NPF, NRT, AMT, PHT, MRS2, NIPA, and ZIP, were significantly downregulated in ‘gold crown’ roots under salt stress; however, they remained constant in ‘jx1502,’ except for an induced NPF gene and a downregulated ZIP gene. Widely downregulated NPF, NRT, AMT, and PHT may lead to a lack and imbalance of nitrogen and phosphorus elements in plants, which may have a serious impact on plant growth and production (Fan et al., 2009; Léran et al., 2014; Bu et al., 2019; Lv et al., 2021). MRS2 family proteins and NIPA have been reported to have a putative function in Mg^2+^ transport and homeostasis (Zhang et al., 2019c; Kobayashi, 2022). Therefore, the downregulation of DEGs encoding these Mg^2+^ transporters in ‘gold crown’ may reflect how salt stress is affected by Mg^2+^ transport. In addition, ZIP family metal transporters are related to iron, zinc, and manganese uptake (Lin et al., 2009; Milner et al., 2013). Notably, three out of four DEGs encoding ZIPs were downregulated by salt stress in ‘gold crown,’ which might lead to a decrease in micronutrient uptake in salt-treated ‘gold crown’ roots. However, the stable expression of genes encoding the above nutrient transporters and the exceptional upregulation of NPF in ‘jx1502’ under salt stress may contribute to its relatively normal nutrient status and growth.

### Different strategies in root growth regulation were identified in ‘jx1502’ and ‘gold crown’ in response to salt stress

The maintenance of lateral root growth is an obvious benefit for the enhancement of water-use efficiency and nutrient uptake, and it serves as an efficient strategy for stress adaptation (Casimiro et al., 2003; Liu et al., 2020c). Previous studies have indicated that lateral root development in response to salt stress can be mediated by plant hormones, such as ABA and IAA (Lu et al., 2019; Van Zelm et al., 2020). For IAA in this process, its dynamic distribution, mediated by three major AUX/LAX influx carriers, PIN efflux carriers, and ABCB/PGP/MDR, plays a crucial role (Titapiwatanakun and Murphy, 2009; Barbez et al., 2012). In this study, genes encoding such IAA transporters were downregulated in ‘gold crown’ in response to salt stress, while they remained constant in ‘jx1502.’ In addition, genes encoding several other proteins that were also required for polar IAA transport and plant adaptability to environmental stimuli were especially upregulated in ‘jx1502,’ including the auxin transport protein BIG and ELP (Nelissen et al., 2010; Cheng et al., 2019). Thus, more active IAA transport in ‘jx1502’ under salt stress may contribute to its better performance in root architecture remodeling and adaptability to environmental stimuli.

In contrast to IAA, ABA produces a negative effect in regulating the growth of lateral root (Duan et al., 2015). It is known that ABA signaling is perceived by the receptor pyrabactin resistance/pyrabactin resistance like (PYR/PYL), which inactivates PP2C, the negative regulator of ABA signaling. Then the activated protein kinase SnRK2 can regulate the expression of certain genes through phosphorylation on transcription factors. Most components of this signaling pathway are required for lateral root growth suppression by ABA (Xing et al., 2016; Liu et al., 2020c). In our data, the DEGs encoding PP2C were upregulated in ‘jx1502’ and downregulated in ‘gold crown.’ The gene encoding PYL was specifically downregulated in ‘jx1502.’ As a result, ‘jx1502’ may obtain a stronger negative regulation capacity for the ABA signal, thus weakening the negative role of ABA on lateral root development. Taken together, the different responses of IAA- and ABA-related genes in the two genotypes to salt stress may contribute to their differences in root system architecture and thus salt tolerance.

Compared to plant hormones, root growth results more directly from cell division and growth. CDC plays an important role in regulating the cell cycle and plant development through the turnover of key proteins *via* ubiquitin-proteasome system degradation, and its mutants result in premature senescence and plant death (Huang et al., 2016). In addition, the transcription factor E2FA induces the genes required for cell cycle progression (Vandepoele et al., 2005). In the present study, DEGs encoding CDK and E2FA were upregulated in ‘jx1502,’ while they were widely downregulated in ‘gold crown.’ A CDC gene was specially downregulated in ‘gold crown.’ Similarly, most DEGs related to cell growth were also induced in ‘jx1502’ but repressed in ‘gold crown.’ For instance, tubulin and actin are key components of the cytoskeleton and play regulatory roles in cell growth (Wasteneys and Galway, 2003). Several DEGs encoding tubulin and MAP were mainly upregulated in ‘jx1502’ and downregulated in ‘gold crown.’ A gene encoding CCT7, which plays a role in the folding of actin and tubulin (Hill and Hemmingsen, 2001), was especially upregulated in ‘jx1502.’ In addition, genes related to actin cytoskeleton reorganization, which is required for cell growth and stress response, were also differentially regulated between two genotypes, such as those encoding protein PIR and ADF4 (Li et al., 2004; Henty et al., 2011), which were upregulated in ‘jx1502’ and downregulated in ‘gold crown,’ respectively. However, as the crucial modulator of cell expansion adaptable to cell division and growth altered by exogenous stimuli, the cell wall is usually in a dynamic changing process, with modifications in structure and the production of its components (Wen et al., 1999). Consistent with the response of cell division and growth in two genotypes, numerous DEGs involved in cell wall construction and modification were identified and were also mainly upregulated in ‘jx1502’ but downregulated in ‘gold crown,’ such as those encoding ELD1, CALS7, and expansin (EXPB1a and EXPA4). Among these, ELD1 can coordinate cell elongation and cellulose synthesis (Pagant et al., 2002). CALS7 plays an important role in phloem development (Barratt et al., 2011; Xie et al., 2011). Expansin has a function in plant growth and development by altering cell wall extensibility, and transgenic plants that overexpress expansin genes display a salt-tolerant phenotype (Han et al., 2012; Lü et al., 2013). Thus, from a cellular perspective, root growth was also reduced in ‘gold crown,’ while it was enhanced in ‘jx1502’ as a possible salt-tolerance trait.

### ‘Jx1502’ enhanced energy supply and compatible solute accumulation for root tolerance

Sugar metabolism and amino acid metabolism are crucial to providing energy and osmotic regulatory substances in response to salt stress (Liu et al., 2020a). It was reported that the increased accumulation of sugar and polyols (sucrose, melibiose, galactinol, sorbitol, and trehalose) from sugar metabolism was related to better growth performance in salt-tolerant plants under salt stress (Widodo et al., 2009; Huang et al., 2018). Similarly, the accumulation of melibiose and galactinol significantly increased in ‘jx1502’ under salt stress, in accordance with the altered expression of related genes in sugar metabolism processes. Moreover, glucose, the most important energy source that contributes to plant growth under salt stress (Wang et al., 2019), was also increased in jx502 by the enhanced hydrolysis of sucrose, starch, and cell wall polysaccharides. The crucial catabolic pathways of glucose, glycolysis, and the TCA cycle are enhanced by the upregulated expression of related DEGs, during which the generated intermediate metabolites and energy are important for plant growth and salt stress defense (Plaxton, 1996; Fernie et al., 2004; Zhong et al., 2015). Therefore, the regulation of DEGs and metabolites in the sugar metabolism processes of ‘jx1502’ may act together to resist salt stress. In contrast, most DEGs related to sugar metabolism in ‘gold crown’ were repressed, consistent with its altered accumulation of metabolites, including sugars, such as glucose, and polyols, such as mannitol and sorbitol, revealing that ‘gold crown’ failed to produce more energy and osmotic regulatory substances to combat salt stress.

Serving as basic elements of proteins, increased levels of amino acids are considered essential for plant salt stress tolerance by maintaining cell membrane stability, improving osmotic regulation, and avoiding oxidative damage (Liu et al., 2020a). Currently, salt stress leads to the increased accumulation of several amino acids in both genotypes. Asparagine can scavenge cytotoxins and protect protein SH groups from oxidation and ROS (Liu et al., 2020a), and it was common in both genotypes and showed greater changes in ‘jx1502,’ which was consistent with the upregulated expression of genes encoding asparagine synthase that catalyzed the synthesis of asparagine from aspartate. In addition, the accumulation of proline, histidine, threonine, arginine, and methionine specifically increased in ‘jx1502,’ and leucine, tyrosine, and glutamine specifically increased in ‘gold crown,’ which was also in accordance with the regulation of genes in complex metabolic processes. For example, the increased accumulation of proline, an important osmoregulation substance (Wu et al., 2017), in ‘jx1502’ under salt stress may be achieved by the upregulated expression of synthesis-related genes encoding delta-1-pyrroline-5-carboxylate synthetase and the downregulated expression of degradation-related genes encoding proline dehydrogenase. In the ‘gold crown,’ the increased accumulation of tyrosine was also achieved by its reduced degradation combined with increased biosynthesis. Similar results were also reported by Huang et al. (2018) and Zhang et al. (2019a), in which the increased accumulation of amino acids generally occurred in both contrasting genotypes differing in salt tolerance under salt stress, with differences in response intensity and amino acid types. Therefore, these results indicate that the increased accumulation of amino acids may be a general response of plants to salt stress, and there is also the possibility that the response intensity and types participate in determining their difference in tolerance.

## Conclusion

In this study, the different responses of ‘jx1502’ and ‘gold crown’ to salt stress were investigated by transcriptome and metabolome analyses. First, ‘jx1502’ induced more transporters and proteins for modulating K^+^/Na^+^ and water homeostasis and maintained a more constant transport system for nutrient uptake and distribution than ‘gold crown’ under salt stress. In addition, ‘jx1502’ strengthens the response to IAA and cell division and growth for root remodeling and thus salt tolerance. Moreover, ‘jx1502’ enhanced sugar and amino acid metabolism for energy supply and osmotic regulatory substance accumulation to meet the demands of protective mechanisms against salt stress.

## Data availability statement

The datasets presented in this study can be found in online repositories. The names of the repository/repositories and accession number(s) can be found below: https://www.ncbi.nlm.nih.gov/, PRJNA871773.

## Author contributions

YC designed this study. XZ, CH, YuqL, YY, and YunL performed the experiments. YC, CH and XZ analyzed the data. YC, and XZ drafted the manuscript. All authors contributed to the article and approved the submitted version.

## Funding

This work was supported by S&D Program of Hebei (22326309D), HAAFS Science and Technology Innovation Special Project (2022KJCXZX-JZS-08), The Earmarked Fund for Hebei Modern Agro-industry Technology Research System (HBCT2021200201,HBCT2021200202), and “Giant Project” of Hebei Province (2018-3).

## Conflict of interest

The authors declare that the research was conducted in the absence of any commercial or financial relationships that could be construed as a potential conflict of interest.

## Publisher’s note

All claims expressed in this article are solely those of the authors and do not necessarily represent those of their affiliated organizations, or those of the publisher, the editors and the reviewers. Any product that may be evaluated in this article, or claim that may be made by its manufacturer, is not guaranteed or endorsed by the publisher.
