# Supplementary material for: Combined full-length transcriptomic and metabolomic analysis reveals the regulatory mechanisms of adaptation to salt stress in asparagus
Source: Front Plant Sci. 2022 Oct 27;13:1050840. doi: 10.3389/fpls.2022.1050840 (PMC9648818; doi:10.3389/fpls.2022.1050840)
Supplement: Supplementary file 1 [file DataSheet_1.zip › Supplementary Figure 1.docx]

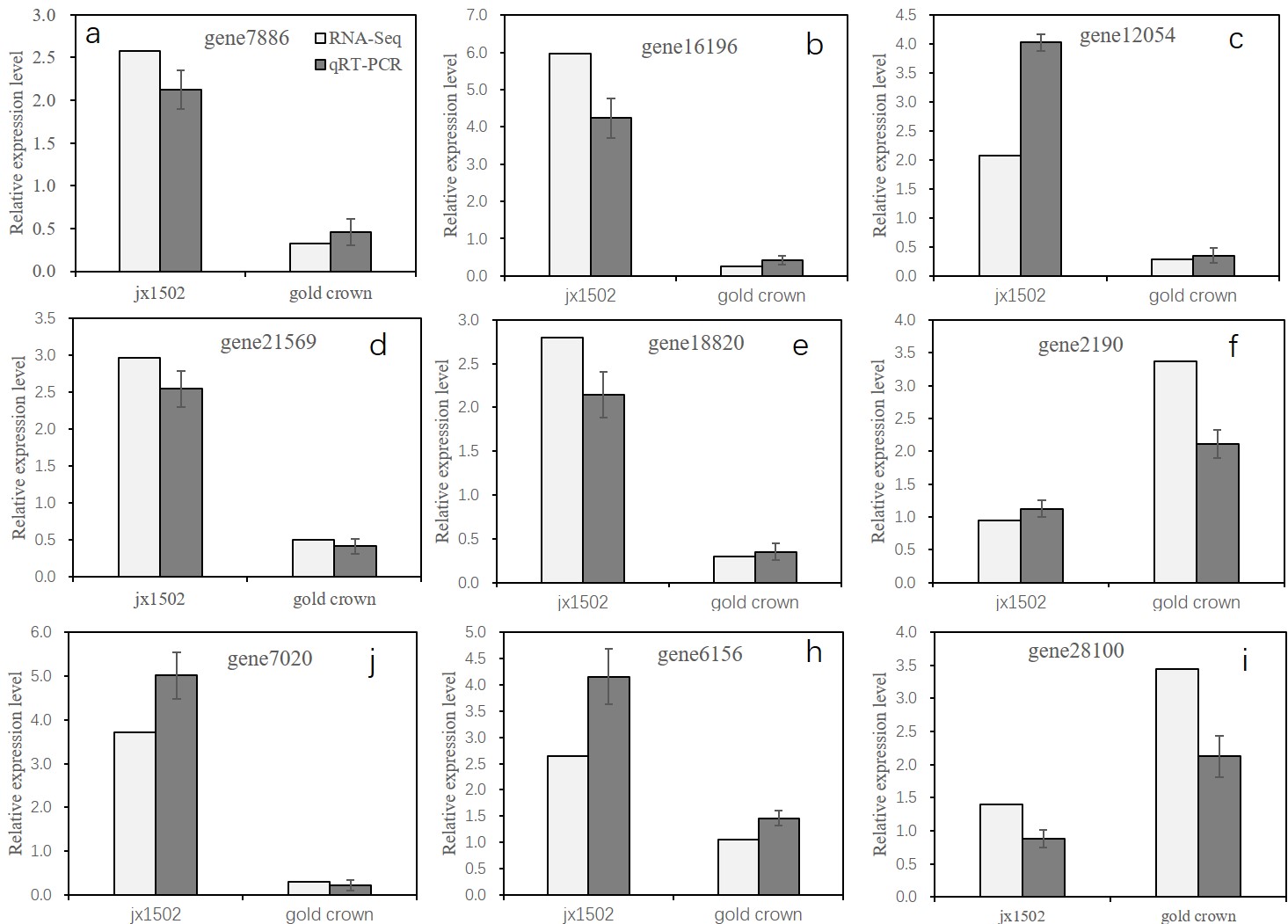


**Supplementary Figure 1** qRT-PCR validation of 9 DEGs. The relative expression level of each gene was expressed as the fold change relative to the control (irrigated with distilled water). (a) *protein NRT1/ PTR FAMILY 2.13 NPF.* (b) *callose synthase 7.* (c) *probable aquaporin NIP5-1.* (d) *tubulin.* (e) *potassium channel KOR1.* (f) *auxin-responsive protein IAA18.* (g) *calcium-transporting ATPase 3.* (h) *cation/H^+^ antiporter 19.* (i) *plasma membrane ATPase.*
